# Neuronal and glial purinergic receptors functions in neuron development and brain disease

**DOI:** 10.3389/fncel.2013.00197

**Published:** 2013-10-28

**Authors:** Ana del Puerto, Francisco Wandosell, Juan José Garrido

**Affiliations:** ^1^Department of Molecular, Cellular and Developmental Neurobiology, Instituto Cajal, Consejo Superior de Investigaciones CientíficasMadrid, Spain; ^2^Centro de Investigación Biomédica en Red de Enfermedades NeurodegenerativasMadrid, Spain; ^3^Centro de Biologïa Molecular “Severo Ochoa”, Consejo Superior de Investigaciones Científicas-Universidad Autónoma de MadridMadrid, Spain

**Keywords:** purinergic receptors, axon, neuron–glia interactions, P2X, P2Y, axon growth

## Abstract

Brain development requires the interaction of complex signaling pathways, involving different cell types and molecules. For a long time, most attention has focused on neurons in a neuronocentric conceptualization of central nervous system development, these cells fulfilling an intrinsic program that establishes the brain’s morphology and function. By contrast, glia have mainly been studied as support cells, offering guidance or as the cells that react to brain injury. However, new evidence is appearing that demonstrates a more fundamental role of glial cells in the control of different aspects of neuronal development and function, events in which the influence of neurons is at best weak. Moreover, it is becoming clear that the function and organization of the nervous system depends heavily on reciprocal neuron–glia interactions. During development, neurons are often generated far from their final destination and while intrinsic mechanisms are responsible for neuronal migration and growth, they need support and regulatory influences from glial cells in order to migrate correctly. Similarly, the axons emitted by neurons often have to reach faraway targets and in this sense, glia help define the way that axons grow. Moreover, oligodendrocytes and Schwann cells ultimately envelop axons, contributing to the generation of nodes of Ranvier. Finally, recent publications show that astrocytes contribute to the modulation of synaptic transmission. In this sense, purinergic receptors are expressed widely by glial cells and neurons, and recent evidence points to multiple roles of purines and purinergic receptors in neuronal development and function, from neurogenesis to axon growth and functional axonal maturation, as well as in pathological conditions in the brain. This review will focus on the role of glial and neuronal secreted purines, and on the purinergic receptors, fundamentally in the control of neuronal development and function, as well as in diseases of the nervous system.

## INTRODUCTION

A plethora of different regulatory molecules are involved in the crosstalk between neurons and glia during neuronal development. In many cases, glial cells secrete molecules that are detected synchronously, either by the neuron as a whole or specifically by the axonal growth cone. Many studies have described the essential role of neurotrophic factors and their tyrosine kinase receptors (nerve growth factor (NGF), brain-derived neurotrophic factor (BDNF), NT-3, FGFs, insulin-like growth factor 1 (IGF-I), etc.) in axon growth and neuronal survival. Indeed, many of these factors are produced by glial cells to modulate neuronal behavior during development. These factors control the activity of PI3-kinase (Alsina et al., 2012;Numakawa et al., 2012), which is essential for axon development, elongation, and maintenance (Sanchez et al., 2001;Shi et al., 2003), and indeed, the activity of this kinase can be regulated through different membrane receptors and adhesion molecules, including integrins.

The insulin/IGF-I system it has been studied widely in both neuronal and non-neuronal cells, controlling processes such as survival-apoptosis (Pap and Cooper, 1998). This survival route is controlled by an insulin-IGF-I-receptor/PI3K/Akt pathway. In addition, central and peripheral insulin-like peptides (ILPs), including insulin, IGF-I, and IGF-II, can produce many other distinct effects in the brain and in neurons (Llorens-Martin et al., 2008;Fernandez and Torres-Aleman, 2012). For instance, the PI3K/Akt pathway appears to regulate neuritogenesis/axonogenesis (Shi et al., 2003;Sosa et al., 2006) and in fact, PI3K inhibition prevents axonal initiation in hippocampal neurons (Shi et al., 2003), or it induces growth cone collapse and neurite retraction (Sanchez et al., 2001), demonstrating the role of PI3K activity in axonal elongation. GSK3 acts downstream PI3K and it represents a second element controlling axonogenesis and neuronal polarity, to the point that GSK3 inhibition (Shi et al., 2004) or GSK3α/β suppression prevents neurons from polarizing (Garrido et al., 2007).

G-protein-coupled receptors (GPCRs) also play an important role in neuronal development and of these, purinergic receptors are important regulators of neuronal development in the context of neuron–glia interaction. The signaling pathways controlled by these GPCRs receptors are not completely deciphered, although it has been demonstrated that they selectively activate different sets of heterotrimeric G proteins. In addition, these GPCRs control neuronal development by acting synergistically, in conjunction with growth factor receptors. While some signaling pathways and trophic factors have been studied extensively during neuronal development, the role of other molecules and their receptors secreted by glia and/or neurons require need further study to fully understand their participation in the modulation of signaling pathways, as is the case of the components of the purinergic system.

## EXPRESSION OF PURINES AND PURINERGIC RECEPTORS IN GLIAL CELLS AND NEURONS

In the nervous system, ATP fulfils a relevant role in the regulation of several physiological functions involving neuron–glia signaling networks. For example, ATP modulates synaptic transmission and a multitude of trophic effects, such as neural cell growth and development. In neurons, ATP is not only released by the pre-synaptic terminal, it can also be released by the post-synaptic membrane (Vizi et al., 1992;Sawynok et al., 1993). In glial cells, several studies show that astrocytes and other glial cells contain the machinery necessary to release ATP (Fields and Stevens, 2000) and there is considerable evidence that glial ATP release is important in glia–glia and neuron–glia communication (for review seeKoles et al., 2011). Moreover, the ATP secreted by neurons and glial cells also contributes to various pathological disorders (Burnstock et al., 2011b), such as hypoxia or other types of brain damage. In fact, millimolar concentrations of ATP can be generated in the extracellular milieu of a cell when it dies, which can activate protective, regenerative and also harmful mechanisms (Cook and McCleskey, 2002;Volonte et al., 2003;Koles et al., 2005;Franke et al., 2006b;Burnstock, 2007).

**FIGURE 1:**
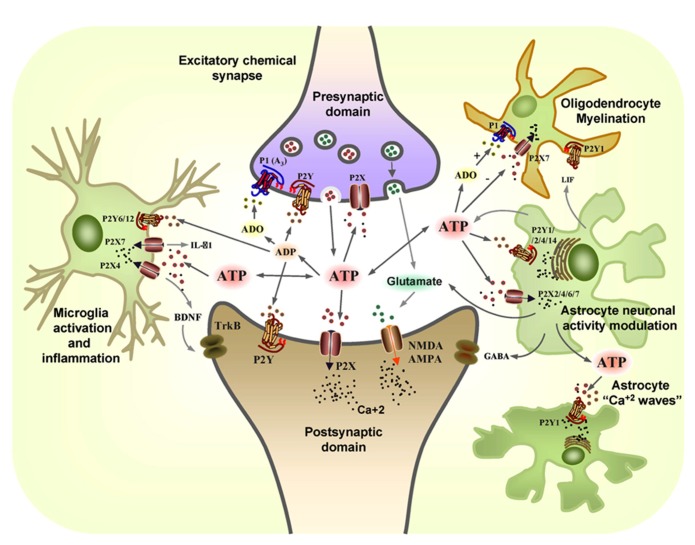
**The coordinated action of glial and neuronal purinergic receptors and purines in the CNS.** Pre-synaptic terminals exocytotically release both glutamate (GLUT) and ATP as co-transmitters. Extracellular ATP is broken down by ectonucleotidases to ADP and adenosine (ADO), with ATP and ADP acting post-synaptically on P2X and P2Y receptors subtypes, while glutamate acts post-synaptically on α-amino-3-hydroxy-5-methyl-4-isoxazole propionic acid receptors (AMPARs) and/or *N*-methyl-D-aspartate receptors (NMDARs). Conversely, the ATP released and its breakdown products, ADP and adenosine (ADO), also act pre-synaptically to modulate neurotransmitter release through the activation of P2X, P2Y, and P1 (A3) receptors. Resting microglia express the P2X4 and P2X7 receptors that are involved in neuropathic pain. ATP promotes IL-1β release through P2X7 receptors and it leads to the release of brain-derived neurotrophic factor (BDNF) through the activation of P2X4 receptors that acts on TrkB receptors expressed by neurons in the pain pathway. P2X7 and P2Y1 receptors mediate the migration of resting microglia after injury, whereas the P2Y6 receptors expressed on the activated microglia mediate phagocytosis at the site of damage. ATP is also released from astrocytes, together with glutamate and GABA, modulating neuronal activity. Leukaemia inhibiting factor (LIF) is released by astrocytes in response to ATP and it promotes myelination by oligodendrocytes through P2Y1 receptors. The P2X7 receptors on oligodendrocytes mediate apoptosis while ADO protects them through the P1 receptor (adapted fromBurnstock, 2008).

These multiple effects of ATP are not only regulated by purinergic receptors but also, by extracellular ectonucleotidases capable of regulating extracellular ATP, ADP, AMP, and adenosine concentrations (Zimmermann et al., 2012). This regulated variation in purine concentrations makes the purinergic system an important mechanism to modulate different activities in neurons and glial cells. Thus, deregulation of the purinergic system can clearly be involved in nervous system pathologies. The large number of purinergic receptors identified and the different signaling pathways modulated by them makes this system particularly complex. This extraordinary combination of factors expands the functional relevance of purinergic signaling (**Figure 1**).

Extracellular nucleotides act through an extended family of nucleotide receptors that can be divided into two families activated by adenosine or ATP/ADP nucleotides, respectively: the P1 and P2 receptors. Four different subtypes of GPC adenosine receptors (P1) have been cloned that are widely expressed in neurons, astrocytes, oligodendrocytes and microglia: A_1_, A_2A_, A_2B_, and A_3_ (Dare et al., 2007;Burnstock et al., 2011a). The P2 receptors are subdivided in two different subfamilies, the ionotropic P2X and the metabotropic P2Y receptors. P2X receptors are ligand gated ion channels whose activation by ATP increases Na^+^, K^+^, and Ca^2^^+^ permeability, promoting rapid changes in membrane potential (North, 2002;Roberts et al., 2006). All seven P2X receptors subtypes (P2X1–7) are expressed by neurons and astrocytes, and some of them are expressed by in oligodendrocytes, Schwann cells, and microglia. They are each involved in different processes, such as fast synaptic transmission, synaptic plasticity, and fast neuronal–glial signaling (Edwards et al., 1992;Silinsky et al., 1992;Pankratov et al., 1998,^2002^,^2009^;Burnstock et al., 2011a;Lalo et al., 2011). By contrast, P2Y metabotropic receptors are related to more long-lasting and trophic functions. Eight P2Y receptors subtypes have been identified and they are activated by different extracellular nucleotides: P2Y1, P2Y12, and P2Y13 are preferentially activated by ADP; P2Y2 and P2Y4 by ATP/UTP; P2Y6 by UDP; P2Y11 by ATP; and P2Y14 by UDP-sugars (Burnstock, 2007). However, the sensitivity of each receptor for a specific nucleotide depends on the mammalian species investigated. Thus, typically, P2Y1, P2Y2, P2Y4, and P2Y6 are coupled to Gq proteins and activate phospholipase C, whereas P2Y12, P2Y13, and P2Y14 couple to Gi proteins, which results in the inhibition of adenylyl cyclases and the reduction of intracellular cAMP (Abbracchio et al., 2006). Most of these receptors are expressed in the nervous system, including neurons and glial cells in central and peripheral nervous system (CNS and PNS), and they are generally considered to be involved in bi-directional neuronal–astroglial communication, exerting long-term effects on proliferation, differentiation, migration, and apoptosis (Neary and Zimmermann, 2009;Verkhratsky et al., 2009;Burnstock et al., 2011b).

Glial cells in the CNS express different purinergic receptors (for an extensive review, seeBurnstock and Knight, 2004). Briefly, CNS astrocytes express P2X1, P2X2, P2X3, P2X4, P2X5, and P2X7 receptors, although only the P2X1/P2X5 and P2X7 receptors have been attributed a functional role (Lalo et al., 2008;Nagasawa et al., 2009). They also express P2Y1, P2Y2, P2Y4, P2Y6, P2Y12, and P2Y14 receptors (Fischer et al., 2009), and functional adenosine receptors (A_1_,A_2A_,A_2B_,A:Brodie et al., 1998;Biber et al., 1999;Trincavelli et al., 2004;Wittendorp et al., 2004). Microglial cells are characterized by the predominant expression of functional P2X4 and P2X7 receptors, yet they also express P2Y1, P2Y2, P2Y2/4, P2Y6, and P2Y12 receptors (Sperlagh and Illes, 2007). Of these, P2Y6 and P2Y12 are functionally relevant to microglia cells (Boucsein et al., 2003;Sasaki et al., 2003;Inoue, 2008). Oligodendrocytes progenitors are characterized by the functional expression of P2X7, P2Y1, P2Y2, P2Y4, P2Y6, P2Y11, and P2Y13 receptors, and there is evidence that they express mRNA encoding all the adenosine receptor subtypes, even though functional expression of only the A1 receptor has been identified in myelinating mature oligodendrocytes (Othman et al., 2003;Agresti et al., 2005a,b;Fields and Burnstock, 2006). Thus, the complexity of the purinergic system is even higher when we take in account the existence of these distinct receptor combinations during development, as well as in physiological and pathological states.

In neurons, P2X3 receptors have been identified in some single rat midbrain synaptic terminals (Diaz-Hernandez et al., 2001), as well as in the central terminals of dorsal root ganglion neurons (DRGs) in the dorsal horn of the spinal cord (Vulchanova et al., 1997). Other P2X receptors, such as P2X2 and P2X4, are widely distributed in neuronal structures, including the cortex, hippocampus, cerebellum, spinal cord, and different brain nuclei (Kanjhan et al., 1999;Norenberg and Illes, 2000;Bo et al., 2003). Moreover, P2X7 receptors have been identified in pre-synaptic terminals (Atkinson et al., 2004) and axonal growth cones (Diaz-Hernandez et al., 2008;del Puerto et al., 2012). There is strong evidence that P2X7 receptors influence neuron activity (Sperlagh et al., 2006;Diaz-Hernandez et al., 2008;Carrasquero et al., 2009;Norenberg et al., 2010;Oliveira et al., 2011;del Puerto et al., 2012), and they may coordinate microglial and neuronal/astroglial responses, both under physiological and pathological conditions (Fields and Stevens, 2000;Ferrari et al., 2006;Sperlagh et al., 2006). With regards the P2Y receptors, P2Y1 receptors are those dominantly expressed in neurons, while P2Y2 receptors are also expressed at lower levels in all regions. In addition, P2Y4, P2Y6, P2Y11, P2Y12, P2Y13, and P2Y14 receptors have been seen to be differentially expressed in several types of neurons (Moore et al., 2001;Verkhratsky et al., 2009).

In the PNS, glial cells and neurons also express different purinergic receptors, the combination of which depends on the neuronal and glial cell type and function (Fields and Stevens, 2000). For example, non-myelinated Schwann cells express A_2A_, A_2B_, P2X7, P2Y1, and P2Y2 receptors, although only P2X7 and P2Y2 receptors are functional in myelinating Schwann cells.

## PURINERGIC RECEPTORS AND PURINES IN NEURON–GLIA INTERACTIONS DURING NEURONAL DEVELOPMENT AND PHYSIOLOGY

The differential expression of purinergic receptors during neuronal and glial development, in combination with the mechanisms that control extracellular purine concentrations, establishes the purinergic system as a global mediator of nervous system plasticity capable of regulating different developmental and functional events, from neurogenesis to neuronal excitability.

### NEUROGENESIS

Different types of glial cells participate in the generation of new neurons in the brain, both during development and at adult stages. These new neurons are generated in neurogenic “niches” that can be considered as functional units of cells, many of which are glial (astrocytes, microglia, etc.), and they are orchestrated by secreted molecules and the extracellular matrix. In the adult rodent brain neurogenesis occurs in two main regions, the subventricular zone (SVZ) of the lateral ventricles and the dentate gyrus of the hippocampus (Altman and Das, 1965;Gage et al., 1998;Alvarez-Buylla and Garcia-Verdugo, 2002). Four types of stem-like cells have been identified in the SVZ: type E cells or ependymal ciliated cells; slow proliferating type B cells (nestin and GFAP positive cells); type C cells or transit amplifying progenitors (nestin positive cells); and proliferating type A neuroblasts. These neurogenic regions are associated with the microvasculature in what is denominated the perivascular niche for neurogenesis (Palmer, 2002). In this region, both angiogenesis and neurogenesis are closely related. Different types of cells in these perivascular neurogenic niches influence the generation and differentiation of new neurons, such as astrocytes, astrocyte-like stem cells, microglia, or endothelial cells, acting through cell–cell interactions, or the secretion of trophic factors, neurotransmitters, hormones, etc. (Doetsch, 2003;Alvarez-Buylla and Lim, 2004;Abrous et al., 2005). These effects are reciprocal and accordingly, neurons contribute to the differentiation of glial cells, such as oligodendrocytes (Song et al., 2002). Moreover, it is possible that the heterogeneity of glial cells in the brain may go some way to explain the variation in different neurogenic regions.

There is now new evidence demonstrating a role for purines and purinergic receptors in the regulation of neurogenesis. It is widely known that extracellular ATP is one of the main regulators of embryonic neurogenesis through the activity of the P2Y1 receptor. In radial glia fibers, P2Y1 activation induces intracellular inositol triphosphate-mediated Ca^2^^+^ release that provokes the liberation of growth factors, ATP, and other neurotransmitters to the extracellular environment (Wiencken-Barger et al., 2007;Elias and Kriegstein, 2008). ATP released from radial glia activates P2Y1 receptors in the neighboring cells, thereby generating calcium waves that extend from the ventricular zone to the pia, expanding across the entire thickness of the embryonic cortex. The formation of these calcium waves between radial glia fibers promotes the proliferation of neuronal progenitors and their synchronization in the S phase of the cell cycle, and indeed, the non-specific purinergic P2 receptor antagonist, suramin, causes a reduction in cell proliferation in the ventricular zone (Weissman et al., 2004). Thus, purinergic signaling represents an important element involved in the maintenance of the neuronal progenitor pool during cortical neurogenesis. Subsequent studies on cultured neurospheres have characterized the purinergic signaling components involved in these events. P2X4, P2X7, P2Ys, and adenosine receptor mRNA has been shown to be expressed in neurospheres (Stafford et al., 2007;Grimm et al., 2009) and moreover, mitotically active neurospheres secrete ATP and undergo purinergic receptor-activated calcium mobilization, suggesting a role for purines and purinergic receptors in the regulation of neuronal progenitor expansion (Lin et al., 2007;Stafford et al., 2007). Adenosine is also implicated in the regulation of neurosphere proliferation in a two ways. While on the one hand the A1 receptor activates MEK/ERK and Akt signaling pathways (Migita et al., 2008), A2a receptor activation inhibits neurosphere proliferation (Stafford et al., 2007).

Purinergic signaling is also coordinated with growth factor signaling during neurogenesis. For example, epidermal growth factor (EGF) activates the same intracellular signaling cues as P2Y1 and P2Y2 receptor agonists (Lin et al., 2007;Stafford et al., 2007;Grimm et al., 2009), suggesting that EGF and purinergic signaling must be coordinated during neurogenesis. Accordingly, cell proliferation is diminished in neurospheres of P2Y1 knockout mice despite the presence of growth factors (Mishra et al., 2006).

ATP release and purinergic signaling may not only be required for developmental neurogenesis but also, for the progenitor cell proliferation that persists in the adult brain. Ectonucleotidase activity is high in the vascular beds subserving both the subventricular and subgranular zones, the two major neurogenic niches in the adult forebrain (Lin et al., 2007). Specifically, the CD39L1 nucleotide triphosphate dinucleotide phosphorylase (NTPDase-2) is expressed in perivascular astrocytes in neurogenic regions, which means that ADP can activate P2Y receptor signaling in the adjacent neuronal precursors (Braun et al., 2003). NTPDase-2 is also expressed in slow proliferating precursor stem cells (type B cells) of the SVZ, in glial tube cells of the rostral migratory stream (RMS;Braun et al., 2003;Langer et al., 2007), as well as in neuronal precursor cells in the hippocampus where it co-localizes with P2Y receptors (Braun et al., 2003;Shukla et al., 2005). Another ectonucleotidase, tissue-non-specific alkaline phosphatase (TNAP), which produces adenosine from extracellular ATP, is expressed by cells of the SVZ and throughout the RMS suggesting a possible role of adenosine receptors in adult neurogenesis (Langer et al., 2007). Finally, functional expression of P2X receptors is detected in hippocampal neural progenitor cells, as reflected by inward currents, membrane depolarization, as well as transient increases in intracellular Ca^2^^+^ concentrations (Hogg et al., 2004;Shukla et al., 2005).

In conclusion, ectonucleotidase signaling can negatively regulate purinergic signaling, clearing ATP in order to prevent uncontrolled expansion of progenitor cells and establishing a permissive microenvironment for neuronal differentiation. Moreover, the association of NTDPase-2 activity with the capillary microvasculature suggests that purinergic signaling may contribute to the angiogenic support of adult neurogenesis (Goldman and Chen, 2011). These data show that the specific regulation of purinergic signaling is crucial for both embryonic neurogenesis during early brain development and to maintain of the neurogenic niches in the adult brain.

### NEURONAL MIGRATION

Neurons born in the ventricular zone of the neural tube populate distant regions of the CNS that are reached by radial and tangential migration (Hatten, 1999). ATP and P2Y1 receptor are involved in the intermediate migration of neuronal progenitors to the neocortical SVZ of developing brain (Liu et al., 2008). It has been shown that the P2Y1 receptor is expressed in cells of the ventricular and SVZ, and that its stimulation with ATP propagates Ca^2^^+^ waves in these cells that can be blocked by the P2Y1 antagonist, MRS-2179 (Liu et al., 2008). Other studies have demonstrated that reduced P2Y1 receptor expression alters the calcium signaling in neural progenitor cells and their migration (Scemes et al., 2003). Migration during adult neurogenesis is also regulated by nucleotides like ATP, ADPβS, or UTP, which in combination with EGF increase focal adhesion kinase (FAK) and Akt phosphorylation in neurospheres isolated from the SVZ. This intracellular signaling contributes to the reorganization of the actin cytoskeleton and drives the migration of neural precursors (Grimm et al., 2010).

### AXON GROWTH

Once neurons or neuronal precursors are generated they must not only migrate to their final destination but they must also extend their axons to contact their targets. Glial cells fulfill an important role in regulating axon growth, both during development and regeneration. Different secreted and extracellular matrix molecules can guide axons and control their growth rate. Indeed, many studies have described essential roles for neurotrophic factors in axon growth and neuronal survival (e.g., NGF, BDNF, NT-3, FGFs, IGF-I, etc.). As mentioned above, these factors control the activity of PI3-kinase, which is essential for axons to develop and elongate. PI3-kinase activity can be regulated through different membrane receptors and adhesion molecules, and recent studies identified a role for purines and purinergic receptors in the modulation of signaling pathways involved in axonal growth, such as that mediated by PI3-kinase.

ATP can be stored and released into the extracellular environment from neurons and glial cells, such as astrocytes, in physiological and pathological conditions (Coco et al., 2003;Pankratov et al., 2006;Thompson et al., 2006;Bowser and Khakh, 2007). In the extracellular environment ATP can be degraded by extracellular ectonucleotidases, which not only control the average half-life of nucleotides but also, they generate new agonists for the different purinergic receptors, such as ADP or adenosine (Zimmermann, 2000). The amount and combination of different purines in the extracellular region during development can regulate the formation and growth of neuronal compartments. During development ATP can be released by different cell types, or liberated after programed or necrotic cell death, generating gradients that can modulate axonal growth and pathfinding. Moreover, large amounts of ATP can be liberated into the extracellular milieu in pathological conditions, impairing regenerative processes in neurons. Our recent studies show that hippocampal neurons express P2X7, P2Y1, and P2Y13 receptors in the distal domain of axons and that ATP acts as a negative regulator of axon growth during axonal elongation, promoting a decrease in axon length in neurons cultured in the presence of ATP. ATP produces an increase of intracellular Ca^2^^+^ in the distal axon of cultured hippocampal neurons, acting through P2X7. The decrease in axon length can be reversed by treating hippocampal neurons with a specific P2X7 antagonist, brilliant blue G (BBG) or using P2X7 interference RNA. Indeed, BBG impaired the ATP dependent increase of intracellular Ca^2^^+^ in the distal region of the axon (Diaz-Hernandez et al., 2008).

By contrast, the product of ATP degradation, ADP, promotes a significant increase in axon length (del Puerto et al., 2012). ADP is the main agonist of three P2Y receptors, P2Y1, P2Y12, and P2Y13. P2Y1 and P2Y13 are expressed in hippocampal neurons (Csolle et al., 2008;del Puerto et al., 2012), whereas P2Y12 has been described in rat brainstem, DRGs (Heinrich et al., 2008), and oligodendrocytes (Amadio et al., 2006), yet it is absent from hippocampal and neocortical neurons (Hollopeter et al., 2001). Although P2Y1 and P2Y13 are activated by the same agonist, they produce opposite effects on axon elongation: P2Y1 is a positive regulator of axonal growth while P2Y13 negatively regulate this process (del Puerto et al., 2012).

These two ADP activated metabotropic receptors, together with the ionotropic P2X7 receptor, share a common signaling pathway that involves type 5 adenylyl cyclase (AC5), and thus, they control of cAMP levels, an important second messenger involved in axon formation and elongation (Shelly et al., 2010). In our model, P2Y1 produces an increase in axon length by activating Gq proteins and AC5, the latter promoting an increase in cAMP in the axonal growth cone, which can be abolished by exposing neurons to the specific AC5 inhibitor, NKY80. By contrast, P2Y13 is coupled to a G_i_ protein that inhibits AC5 activity, thereby decreasing the cAMP concentration in the axon growth cones and generating shorter axons (del Puerto et al., 2012). Finally, AC5 is inhibited by submicromolar concentrations of Ca^2^^+^ (Willoughby and Cooper, 2007), such as those produced by P2X7 activation, thereby provoking negative effects on axon elongation (del Puerto et al., 2012).

**FIGURE 2:**
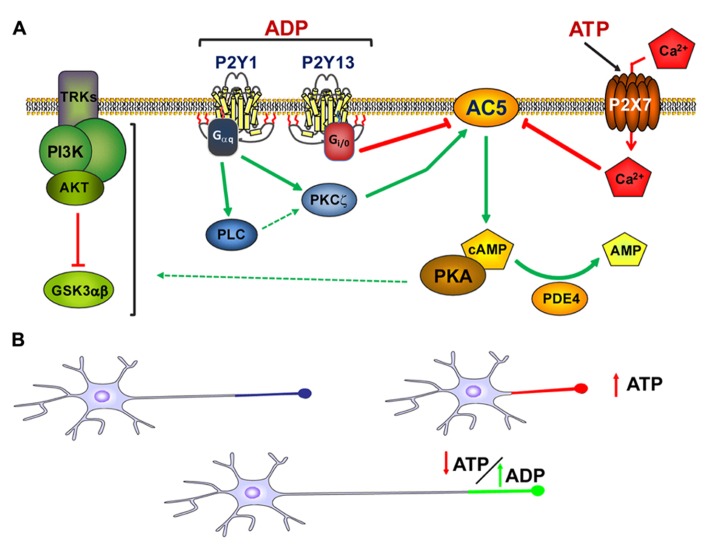
**Modulation of axon growth by purinergic receptors. (A)** Schematic representation of the coordination of purinergic receptors and purines during the modulation of axon elongation in hippocampal neurons. Both ADP acting on P2Y1 and P2Y13 receptors, and ATP acting through P2X7 receptors, modulate adenylyl cyclase type 5 activity (AC5) in a coordinated manner. This coordinated signaling through AC5 serves to modulate cAMP intracellular concentrations and the activity of the PI3K-Akt-GSK3α/β signaling pathway, the latter controlling axon elongation in hippocampal neurons (adapted fromdel Puerto et al., 2012). **(B)** Axon elongation depends on the extracellular concentration of ATP. High extracellular concentrations of ATP provoked by cell damage, programed apoptosis, or other physiological and/or pathological conditions diminish axon elongation. However, in the absence of high extracellular concentrations of ATP, ADP can trigger P2Y1 receptors and promote axon elongation. The balance between the extracellular concentrations of purines and the activity of purinergic receptors modulates axonal elongation.

This coordinated signaling through AC5 serves to modulate one of the main pathways that controls neuronal polarity and axonal elongation, the PI3K-Akt-GSK3α/β signaling pathway (Shi et al., 2003). Modulating P2Y1, P2Y13, and P2X7 activity induces changes in PI3K activation, and modifying Akt and GSK3α/β phosphorylation, and promoting or inhibiting axon growth (Garrido et al., 2007;del Puerto et al., 2012). Thus, extracellular nucleotides released in physiological and pathological conditions can act in a coordinated way through purinergic receptors expressed by neurons to modify axon growth, promoting the arrival of axons to their target to form synaptic contacts (**Figure 2**).

## ROLE OF GLIAL CELLS IN THE GENERATION OF FUNCTIONAL AXONAL DOMAINS

After axons have grown and reached their targets, glial cells contribute to neural activity by enveloping and myelinating axons, generating nodes of Ranvier and their adjacent structures, and providing an electrical insulation for the rapid conduction of nerve impulses. This role is achieved by oligodendrocytes in CNS and Schwann cells in PNS. Axon ensheathing depends on bi-directional signals between myelinating glia and neurons, whereby neuronal activity leads to the release of soluble factors that are detected by myelinating glia. Conversely, myelinating glia interact with axons and thereby communicating with neurons. This interaction is maintained by molecules in the membranes of both cell types, which are differentially distributed in paranodes, juxtaparanodes, and nodes of Ranvier, and that can modulate signaling cascades and specifically concentrate ion channels at nodes of Ranvier or the axon initial segment (AIS). For example, L1 homophilic interactions between axons and Schwann cells are essential for the initiation of myelination and the expression of these molecules can be regulated by neuronal activity (Seilheimer et al., 1989;Wood et al., 1990). In fact, impairing sodium-dependent action potentials with tetrodotoxin (TTX), inhibits axon myelination in an “*in vitro*” model of cultured glial cells and neurons, as occurs in the developing optic nerve after intravitreous injection of TTX. By contrast, the α-scorpion toxin-mediated increase in neuronal firing enhances myelination (Demerens et al., 1996). Different adhesion molecules and growth factors have been proposed to act as intermediate molecules between neuronal activity and myelination, although many details of the mechanisms regulating myelination remain uncertain.

Regarding purinergic signaling, studies in DRG neurons show that neuronal activity releases ATP from premyelinated axons, which can be detected by several purinergic receptors in myelinating glia. ATP inhibits differentiation and myelination by Schwann cells through the activation of P2Y receptors on Schwann cells (Stevens and Fields, 2000). As purinergic receptors expression is developmentally regulated, this inhibition of Schwann cells differentiation and myelination may help to coordinate Schwann cell development with functional activity in the nervous system, thereby preventing premature Schwann cell differentiation. However, another purine, adenosine, stimulates differentiation of oligodendrocyte precursors and myelination in the CNS. These differential effects of purines in the PNS and CNS are probably due to differential expression of purinergic receptors, as well as that of related trophic factors and adhesion molecules. To understand the role of purinergic signaling in myelination, it is necessary to clearly identify which receptors are expressed in myelinating glial cells at each developmental stage. This will also help understand which of these receptors are involved in demyelinating diseases. For example, the P2X7 ATP receptor seems to be involved in the myelin defects associated with experimental autoimmune encephalomyelitis (EAE) or Charcot–Marie–Tooth (CMT) disease (Sharp et al., 2008;Nobbio et al., 2009). Moreover, a molecule able to regulate P2X7 expression, like retinoic acid (Wu et al., 2009), can also regulate myelination (Latasa et al., 2010;Huang et al., 2011). All these data support an important role of purines and purinergic receptors in functional axonal maturation, and they emphasize the importance of future studies regarding the role of purinergic receptors in axon development and the functional maturation of axonal domains, like the nodes of Ranvier or AIS.

### NEURONAL ACTIVITY

In the developing nervous system, neuronal activity plays a major role in neuronal development, regulating axonal pathfinding, the refinement of topographic maps, dendrite morphogenesis, and the segregation of axonal terminal arbors. Glial cells participate in all these processes and modulate neuronal activity. As mentioned above, ATP is released by neurons and astrocytes (Fields and Stevens, 2000) and this extracellular ATP can either bind directly to P2 receptors, or it may be processed by ectonucleotidases in the extracellular space to generate other purines, such as ADP or adenosine. These purines each have specific affinities for different purinergic receptors. For example, P2Y receptors show ATP sensitivity at nanomolar concentrations, while P2X7 receptors require micromolar concentrations of this purine (Surprenant, 1996), giving ATP signaling a very dynamic range of activities. ATP release from astrocytes was initially found in culture systems (Queiroz et al., 1997) and its physiological significance was deduced from the analysis of propagating glial Ca^2^^+^ waves (Arcuino et al., 2002). Thus, this ATP release is able to modulate neuronal activity (Koizumi et al., 2003).

Initial experiments showed that ATP depolarizes neurons (Jahr and Jessell, 1983;Krishtal et al., 1983), subsequently opening single ion channels (Kolb and Wakelam, 1983;Benham and Tsien, 1987), and mediating synaptic transmission at synapses in both the PNS and CNS (Jansen et al., 1990;Edwards et al., 1992;Evans et al., 1992;Silinsky et al., 1992). For example, ATP receptor activation potentiates a voltage-dependent calcium channel in CA3 hippocampal neurons (Dave and Mogul, 1996). Similarly, P2Y1 receptors mediate the activation of neuronal calcium-dependent potassium channels (Schicker et al., 2010), while different P2Y receptors can inhibit two-pore potassium (K(2P)) channels (Shrestha et al., 2010), K_v_7 potassium channels (Hernandez et al., 2008), and Ca_v_2 calcium channels (Filippov et al., 2010). Thus, purinergic signaling is an important modulator of neuronal activity and as such, it will be important to study and understand the intracellular molecular mechanisms that control neuronal excitability through purinergic signaling. Large amounts of ATP seem to be released after an action potential (Richler et al., 2008) that binds to extrasynaptic P2X receptors expressed at dendritic spines and nerve terminals (Vulchanova et al., 1996;Le et al., 1998;Rubio and Soto, 2001). This action of ATP is neuromodulatory but no evidence has been found for a role of ATP in generating action potentials. Most evidence of the neuromodulatory activity of purinergic receptors and purines has come from the PNS. However, pre-synaptic P2X responses have now been described in many parts of the brain and for example, it has been shown that P2X4 may play a role in fast synaptic transmission or in the modulation of neurotransmitter release (Norenberg and Illes, 2000;Rubio and Soto, 2001).

## THE RELATIONSHIP BETWEEN GLIAL AND NEURONAL CELLS IN NERVOUS SYSTEM PATHOLOGIES

Deregulation of physiological of purines and purinergic receptors functions in neurons and glial cells have been described in multiple pathologies in central and peripheral nervous system, including psychiatric and neurodegenerative diseases, and brain damage due to trauma or ischemia.

### EPILEPSY

Extracellular nucleotides and purinergic receptors are involved in epileptic seizures. In the hippocampus of different animal models of epilepsy an increase in the expression and activity of different extracellular ectonucleotidases has been described, such as NTPDase 2 and 3, and ecto-5′-nucleotidase (Schoen et al., 1999;Oses et al., 2004;Cognato Gde et al., 2007), as well as an increase in the extracellular concentration of ATP (Wieraszko and Seyfried, 1989), which would be rapidly broken down to adenosine. In fact, the hydrolysis rates of ATP, ADP, and AMP is significantly enhanced in the serum of patients following an epileptic event (Grosso et al., 2009). Some studies propose that an increase in the density of A1 receptors in the cortex and hippocampus may underlie a reduction in the seizures provoked by chronic NMDA receptor stimulation by glutamate released from astrocytes (Von Lubitz et al., 1995;Tian et al., 2005). On the other hand, astrocytes also regulate the endogenous anticonvulsant effects mediated by adenosine in the brain, since synaptic levels of adenosine are controlled by an astrocyte-based adenosine cycle in which a key element is the activity of the enzyme that removes adenosine, the adenosine kinase (ADK;Etherington et al., 2009). In addition to the involvement of adenosine in epileptic seizures, the role of ATP and ADP has also been studied. Indeed, the amount of extracellular ATP detected in hippocampal slices following electrical stimulation of Schaffer collaterals is significantly greater in mice with inherited susceptibility to seizures than in those that are resistant (Wieraszko and Seyfried, 1989).

It has also been shown that P2X7 receptors are involved in epileptic seizures since the hippocampus of chronic epileptic rats respond abnormally to ATP in association with an increase in the expression of this receptor. P2X7 is also up-regulated, probably in microglia, and it is involved in the inflammatory reaction of epilepsy and may participate in the pathophysiology of temporal lobe epilepsy (Vianna et al., 2002). In fact, kainate-induced seizures result in an elevation of the levels of the P2X7 receptor on microglia as they become activated (Rappold et al., 2006). Moreover, significantly elevated P2X7 immunoreactivity in amoeboid or phagocytoyic microglia appeared in the dentate gyrus 7 days after status epilepticus (Kim et al., 2009). In conclusion, extracellular nucleotides are involved in the modulation of epilepsy and seizures, and they contribute to the activation of purinergic receptors on both astroglial cells and microglia in the epileptic brain, affecting neuronal function.

### BRAIN TRAUMA, HYPOXIA, AND STROKE

It has well documented that mechanical trauma or metabolic limitation, such as trauma, ischemia and stroke, results in an immediate, irreversible loss of tissue at the lesion site, as well as secondary expansion of tissue damage over time. This type of injury promotes the release of ATP/adenosine from different cells aggravating the neuronal and glial damage in the surrounding zone. “*In vivo*” studies using models of focal ischemia, as well as, different models of mechanical injury to the rat nucleus accumbens and the spinal cord, described sustained high release of ATP and glutamate in the peri-traumatic area (Wang et al., 2004;Melani et al., 2005;Franke et al., 2006a;Frenguelli et al., 2007). During ischemia and mechanical injury the ATP released is sufficient to stimulate P2X7 receptors and to kill neurons, an event which can be blocked by P2X7 antagonists like BBG (Arbeloa et al., 2012). Indeed, BBG also promotes the recovery of motor function after mechanical injury to the rat spinal cord (Wang et al., 2004;Peng et al., 2009) and reduces the secondary damage in the mechanical injured brain (Kimbler et al., 2012). Nevertheless, extracellular ATP can also modify synaptic transmission in neurons, affecting their viability. Extracellular ATP released from damage cells can stimulate the pre-synaptic neuronal P2X receptors, promoting enhanced excitatory synaptic transmission (Rodrigues et al., 2005;Zhang et al., 2006;Sperlagh et al., 2007). Meanwhile, activation of P2X7 receptors by ATP in astrocytes may not only permit the release of ATP but also, that of glutamate and GABA in the peri-traumatic area (Pannicke et al., 2000;Wang et al., 2002;Duan et al., 2003). Hence, the hypoxic release of ATP may exert both excitatory and inhibitory effects on neurons, aggravating or diminishing the metabolic damage exerted on neurons and astrocytes.

P2Y1 expression is also enhanced around necrotic tissue, in the peri-traumatic area of the rat cortex and nucleus accumbens after mechanical injury, and in the pre-infarct region after middle cerebral artery occlusion (MCAO;Franke et al., 2004b). In fact, a non-selective P2 receptor antagonist, PPADS, improved the morphological and functional alterations provoked by the ischemic injury (Lammer et al., 2006), leading to a decrease in the infarct volume and reducing neuronal and astrocyte death for up to 7 days after MCAO (Lammer et al., 2011). Other studies have shown that intra-cerebroventricular administration of the P2Y1 agonist, MRS-2365, increases cerebral infarct volume after 72 h of transient MCAO, whereas the P2Y1 antagonists MRS-2179 and MRS-2279 reduced this volume, aiding the recovery of motor coordination (Kuboyama et al., 2011). As mentioned previously, P2Y1 receptors are expressed by astrocytes and they can protect against hydrogen peroxide-induced damage (Fujita et al., 2009). Cytotoxic edema and the magnitude of ischemic lesions are significantly reduced by treatment with a P2Y1 agonist, 2-MeSADP), and this protection is achieved by enhanced astrocyte mitochondrial metabolism due to increased inositol trisphosphate-dependent Ca^2^^+^ release (Zheng et al., 2010). However, inhibition of astrocyte P2Y1 receptors can also result in cytokine/chemokine transcriptional suppression, involving the NF-kB pathway, and brain protection (Kuboyama et al., 2011). The P2Y1 receptor is also thought to play a role in the production of GFAP and GDNF in astrocytes under transient MCAO (Sun et al., 2008), and it participates in astrogliosis (Franke et al., 1999,^2001^,2004b,^2009^). This is due to P2Y1-mediated modulation of PI3K/AKT and MAPK/ERK signaling in astrocytes and neurons, resulting in astroglial proliferation and anti-apoptotic processes (Franke et al., 2009). A protective effect of the A2A receptor agonist has been described in mechanical injured spinal cord and during ischemia, most probably due to the reduction of glutamate outflow from glial cells (Popoli et al., 2003;Pedata et al., 2007). However, A2A receptor inhibition could be protective in a model of permanent focal ischemia, probably by attenuating microgliosis and the production of pro-inflammatory cytokines (Chen and Pedata, 2008). Among these glial cells, microglia are activated by ATP released after mechanical trauma or metabolic limitation and stimulation of P2X7 in microglia is deleterious in cultured neurons as a consequence of oxidative stress, although this effect is halted in microglia that lack this receptor subtype (Skaper et al., 2006). In fact, P2X7 expression and activation in microglia after mechanical injury or metabolic limitation in the penumbra surrounding the necrotic region, is an early reaction, followed by the appearance of these receptors in astroglia and at pre-synaptic elements of neurons (Franke et al., 2004a,^2007^).

Hypoxic ischemic injury also affects oligodendrocytes and the white matter. In general, the ATP released from damage cells during the ischemic process facilitates P2X7 activation in oligodendrocytes, promoting the inward currents and cytosolic Ca^2^^+^ overload that lead to oligodendrocyte death (Domercq et al., 2010). In contrast to what occurs in astrocytes and neurons, hypoxic/ischemic insults might down-regulate P2X7 expression in cultured oligodendrocytes (Wang et al., 2009). Thus, activation of purinergic receptors by ATP released after trauma leads to late responses of glial cells, such as astroglia proliferation, microglia activation, and demyelination in the white matter, which modifies neuronal transmission and viability during these pathologies.

### MULTIPLE SCLEROSIS AND AMYOTROPHIC LATERAL SCLEROSIS

Demyelination in multiple sclerosis (MS) and amyotrophic lateral sclerosis (ALS) involves purinergic receptors signaling, since the nucleotides released in large quantities under inflammatory conditions and following cell death are important mediators in demyelinating diseases. Up-regulation and activation of the A1 adenosine receptor attenuates neuroinflammation and demyelination during chronic EAE, a model of MS (Tsutsui et al., 2004). This effect is related to pro-myelinating effect of A1 receptors in oligodendrocyte precursors. ATP signaling is also implicated in this pathology through the activation of the P2X7 receptor in oligodendrocytes that causes excitotoxicity. Indeed, treatment of chronic EAE models with a P2X7 antagonist reduces demyelination and ameliorates the associated neurological symptoms protecting oligodendrocytes from death (Matute et al., 2007). In fact, P2X7 null mice do not develop EAE associated symptoms (Sharp et al., 2008).

In post-mortem sections of the cerebral cortex from MS patients, the P2Y12 receptor is present in myelin and interlaminar astrocytes but absent from demyelinated axons. Decreased P2Y12 receptor immunoreactivity in the proximity of the lesions is directly correlated with the extent of demyelination (Amadio et al., 2010), suggesting that the loss of purinergic P2Y12 receptors might be detrimental to tissue integrity. A marked increase of P2X7 immunoreactivity in reactive astrocytes has also been observed in brain tissues from MS patients (Narcisse et al., 2005), as well as in microglial cells/macrophages in affected regions of tissue from MS and ALS patients (Yiangou et al., 2006). Astrocytes, infiltrating cells of the monocyte/macrophage lineage and activated microglia can release ATP and thereby contribute further to P2X7 activation and cell death in oligodendrocytes (Matute et al., 2007;Matute and Cavaliere, 2011).

With regards purinergic signaling and ALS, an up-regulation of P2X4, P2X7, and P2Y6 receptors has been observed in transgenic mice over-expressing human superoxide dismutase 1 (SOD1), an animal model of ALS (D’Ambrosi et al., 2009), as well as the down-regulation of ATP-hydrolyzing activities in microglia, suggesting that the pro-inflammatory actions of microglial P2 receptors are enhanced in this ALS model. During the progression of ALS, microglia, astrocytes, and motor neurons might enter in crosstalk via ATP release/degradation and P2X7 activation, generating a feedback loop that drives the sustained pro-inflammatory and detrimental response (Amadio et al., 2011;Volonte et al., 2011) which is prevented by P2X7 antagonists (D’Ambrosi et al., 2009;Gandelman et al., 2010). However, P2X4 receptors exert protective effects in motor neurons. In function of these data, low ATP concentrations protect cells against exocytotoxic stimuli through P2X4 receptors, whereas high concentrations of ATP produce toxic P2X7 activation. Finally, adenosine is also involved in ALS since adenosine A2A receptor antagonists prevent motor neuron death (Mojsilovic-Petrovic et al., 2006).

### ALZHEIMER’S DISEASE

Recent studies have implicated purinergic receptors in neurodegenerative diseases. For example, there is evidence of the involvement of purinergic receptors in Alzheimer’s disease (AD), and A1 receptor expression is lost or reduced in the outer layers of hippocampal dentate gyrus in human brain tissue from AD patients (Jansen et al., 1990;Ulas et al., 1993), while the expression of A1 and A2A receptors appears to be increased in the frontal cortex (Albasanz et al., 2008). With regards the involvement of P2 receptors in this pathology, an up-regulation of the P2X7 receptor occurs around Aβ plaques in a mouse model of AD (Parvathenani et al., 2003) and enhanced P2X7 expression is detected in brain samples from AD patients, especially in microglia associated with Aβ plaques (McLarnon et al., 2006). Moreover, cultured fetal human microglia cells exposed to the amyloidogenic Aβ_1-42_ peptide have elevated levels of P2X7 receptors and an increased amplitude in their Ca^2^^+^ response, which can be abrogated by P2X7 inhibitors (McLarnon et al., 2006). For instance, acting through P2X7 receptors, extracellular ATP can alter β-amyloid peptide-induced cytokine release from macrophages and microglia, making this receptor subtype an important modulator of neuroinflammation in AD (Rampe et al., 2004;Sanz et al., 2009). Finally, the up-regulation of P2X7 and in the production of reactive oxygen species (ROS) in microglia occurs in parallel with the increase in Aβ, and in relation to the synaptotoxicity and cerebral damage in an AD model (Lee et al., 2011). The involvement of the P2X7 receptor in AD is corroborated by the fact that Aβ deposition in an AD mouse model can be prevented by P2X7 antagonists (Diaz-Hernandez et al., 2012). Thus, the P2X7 receptor mediates purinergic inflammatory responses in the AD brain through its activation in microglial cells.

Other P2 purinergic receptors have been involved in AD. Aβ induces a caspase-mediated cleavage of P2X4 receptor in primary rodent neurons. This P2X4 levels reduction attenuates Aβ_1__-__42_-induced neuronal death, while increased P2X4 expression in a neuronal cell line enhances Aβ_1__-__42_ toxic effect (Varma et al., 2009). P2Y2 density reduction is correlated with lower synaptophysin immunoreactivity in post-mortem parietal cortex samples from AD patients (Lai et al., 2008). In addition, P2Y1 receptors have been also localized in characteristic AD structures, such as neurofibrillary tangles, neuritic plaques, and neuropil threads (Moore et al., 2000). Purinergic receptors also contribute to the AD pathology acting on astrocytes. For example, ATP and glutamate released from Aβ_25__-__35_ activated astroglial cells are able to activate neural hemichannels that causes neural damage (Orellana et al., 2011). In the same way, P2X7 receptors activation in mouse primary astrocytes stimulates the non-amyloidogenic APP processing by α-secretases and reduces amyloid plaques (Delarasse et al., 2011;Diaz-Hernandez et al., 2012).

### NEUROPATHIC PAIN

Communication between neurons and surrounding glial cells is implicated in chronic pain and in fact, neuronal–glia communication through purinergic signaling is also involved in neuropathic pain. Adenosine contributes to analgesia due to the combined action of A1 receptor-mediated antinociception and A2A receptor-mediated anti-inflammatory activity (Ledent et al., 1997;Johansson et al., 2001;Bura et al., 2008). The number of microglia and astrocytes is enhanced in wild-type mice subjected to sciatic nerve injury that causes neuropathic pain, and this response is attenuated in A2A receptor knockout animals (Bura et al., 2008). In addition, ATP and P2 receptors have been studied extensively in neuropathic pain. ATP release from Ca^2^^+^ wave propagating spinal astrocytes could play an important role in the conduction of nociceptive information, since DRGs respond to ATP by depolarization and glutamate release (Gu and MacDermott, 1997). Administration of ATP produces long-lasting allodynia, probably via P2X2/P2X3 receptors (Nakagawa et al., 2007) and it has been suggested that P2X3/P2X2/3 receptor-dependent cytosolic phospholipase A_2_ (cPLA_2_) activity in primary sensory neurons is a key event in neuropathic pain (Tsuda et al., 2007). The peripheral equivalents of astrocytes, satellite glial cells, are located in sensory ganglia and they express functional P2X7 receptors (Zhang et al., 2005) that can be stimulated by ATP released from DRGs, in turn causing the release of TNF-α and potentiating P2X3 receptor-mediated responses in nearby neurons (Zhang et al., 2007). Several studies have implicated microglial P2X4 receptors in tactile allodynia and hyperalgesia, contributing to the pain states. P2X4 receptor-immunoreactivity is enhanced in spinal microglia after peripheral nerve injury and intraspinal application of P2X4 receptor antagonists suppresses tactile allodynia (Tsuda et al., 2003). Signaling between microglia and neurons is therefore an essential link in neuropathic pain transmission after peripheral nerve injury. ATP-stimulation of microglia through the P2X4 receptor signals to lamina I neurons and change their phenotype, causing a collapse of their transmembrane anion gradient. In addition, BDNF released from microglia after ATP stimulation provides a crucial signal to neurons during this process (Coull et al., 2005;Keller et al., 2007). P2X7 activation promotes inflammation and neuropathic pain (Donnelly-Roberts and Jarvis, 2007). Indeed, blocking P2X7 receptors provokes anti-nociception that may be explained by the impairment of pro-inflammatory IL-1β release from macrophages or microglia, and by the absence of nociceptor activity in peripheral tissues or pain-transmitting neurons in the CNS (Clark et al., 2010). In addition, IL-1β transiently enhances P2X7 receptor expression and activity in human astrocytes, forming a positive feedback loop (Narcisse et al., 2005).

In terms of P2Y metabotropic receptors, P2Y1 receptors decrease the intensity of pain by blocking voltage-sensitive Ca^2^^+^ channels in the central terminals of sensory neurons within the dorsal horn of the spinal cord and by decreasing the glutamate release from DRG terminals (Gerevich et al., 2004). Recent studies showed that the P2Y13 receptor can counteract the neuropathic effect of the P2Y1 receptor (Malin and Molliver, 2010). However, the most studied P2Y receptor in terms of the pathology of neuropathic pain is P2Y12, the mRNA expression of which is enhanced in microglial cells in the spinal cord ipsilateral to the injured nerve after lumbar nerver injury, a model of neuropathic pain (Tozaki-Saitoh et al., 2008). In addition, activation of P2Y12 receptors induces the release of pro-inflammatory cytokines that induce neuropathic pain, such as IL-1β and TNF-α (DeLeo and Yezierski, 2001). P2Y12 receptors are also required for the extension of microglial processes to mediate a rapid microglial response to injury (Davalos et al., 2005) and the blockade of this receptor subtype with specific antagonists suppresses the increase in the number of microglia attached to myelinated axons, preventing the demyelination process, as well as the development of tactile allodynia (Ando et al., 2010).

## CONCLUDING REMARKS

Purinergic receptors are expressed in all cell types in the CNS and PNS, and they are involved in a complex system of cell signaling. The combination of several purines, multiple types of purinergic receptors, and ectonucleotidases open the possibility of exerting a fine regulation of neuronal and glial activities, and of coordinating these in distinct physiological and pathological states. While purinergic receptors have been studied extensively in glial cells, mainly astrocytes, and microglia, our knowledge about their influence on neuronal function and development still remains unclear. Accordingly, it will be important to understand how neurons respond to purines released by other neurons or glial cells. Thus, future studies into purinergic receptor expression in neurons and their influence on neuronal growth and excitability will help us understand the role of neuron–glia communication in nervous system physiology, as well as aiding the development of therapeutic strategies adapted to specific receptors and cell types.

## Conflict of Interest Statement

The authors declare that the research was conducted in the absence of any commercial or financial relationships that could be construed as a potential conflict of interest.
